# Secondary bacterial infection in COVID-19 patients is a stronger predictor for death compared to influenza patients

**DOI:** 10.1038/s41598-021-92220-0

**Published:** 2021-06-16

**Authors:** Noa Shafran, Inbal Shafran, Haim Ben-Zvi, Summer Sofer, Liron Sheena, Ilan Krause, Amir Shlomai, Elad Goldberg, Ella H. Sklan

**Affiliations:** 1 Department of Medicine D, Rabin Medical Center, Beilinson Hospital, Petah-Tikva, Israel; 2 Department of Internal Medicine II, Medical University of Vienna, Vienna, Austria; 3 Microbiology Laboratory, Rabin Medical Center, Beilinson Hospital, Petah-Tikva, Israel; 4 Department of Clinical Microbiology and Immunology, The Sackler Faculty of Medicine, Tel Aviv University, 6997801 Tel Aviv, Israel; 5 Department of Medicine F, Rabin Medicine, Tel Aviv University, Tel Aviv, Israel; 6 The Sackler Faculty of Medicine, Tel Aviv University, Tel Aviv, Israel

**Keywords:** Infectious-disease epidemiology, Bacterial pathogenesis, Bacterial infection, Viral infection

## Abstract

Secondary bacterial infections are a potentially fatal complication of influenza infection. We aimed to define the impact of secondary bacterial infections on the clinical course and mortality in coronavirus disease 2019 (COVID-19) patients by comparison with influenza patients. COVID-19 (n = 642) and influenza (n = 742) patients, admitted to a large tertiary center in Israel and for whom blood or sputum culture had been taken were selected for this study. Bacterial culture results, clinical parameters, and death rates were compared. COVID-19 patients had higher rates of bacterial infections than influenza patients (12.6% vs. 8.7%). Notably, the time from admission to bacterial growth was longer in COVID-19 compared to influenza patients (4 (1–8) vs. 1 (1–3) days). Late infections (> 48 h after admission) with gram-positive bacteria were more common in COVID-19 patients (28% vs. 9.5%). Secondary infection was associated with a higher risk of death in both patient groups 2.7-fold (1.22–5.83) for COVID-19, and 3.09-fold (1.11–7.38) for Influenza). The association with death remained significant upon adjustment to age and clinical parameters in COVID-19 but not in influenza infection. Secondary bacterial infection is a notable complication associated with worse outcomes in COVID-19 than influenza patients. Careful surveillance and prompt antibiotic treatment may benefit selected patients.

## Introduction

Secondary bacterial infections are a known complication of viral respiratory infections, often leading to clinical deterioration. Secondary bacterial infections were a significant cause of morbidity and mortality in previous well-studied influenza pandemics and during seasonal influenza, as well as in other respiratory diseases^1,2^. Mechanistically, disruption of airway epithelium and its barrier function due to viral-induced immune-mediated damage, and dysregulation of both, the innate and adaptive immune responses are thought to promote the colonization of various bacteria^3,4^.

Despite the clinical importance of secondary bacterial infections, their contribution to COVID-19 severity and mortality is still not well established. Several studies addressed this issue, but variations in testing methodologies, site-specific nosocomial infections, different definitions of early vs. late infections, and differences in treatment modalities, complicate the analysis of these data^5,6^.

Influenza and COVID-19 have different disease courses, with influenza having a shorter incubation time and disease duration^7^. These differences might reflect disparities in the magnitude and kinetics of the immune response to these pathogens, affecting the type and time of the appearance of secondary bacterial infections, as well. Here we compared early (< 48 h after admission) or late (> 48 h after admission) secondary bacterial infections identified in blood or sputum cultures of admitted influenza or COVID-19 patients from a single large tertiary center in Israel.

## Results

### Characterizations of influenza and COVID-19 patients

A total of 1384 admitted patients with available cultures were assessed. Of these, 642 had a confirmed SARS-CoV-2 infection (59.2% males, median age 68 (interquartile range (IQR) 53–78)), while 742 had a confirmed influenza infection (47% males, median age 67 (IQR 43–78)). Patients’ clinical and demographic characteristics are shown in Table 1. Laboratory tests known to correlate with inflammation were more severe among COVID-19 patients compared to influenza patients, specifically, maximal C-reactive protein (CRP) levels (median 13.41 (IQR (6.22–24.94) vs.7.05 (3.3–15.4), p < 0.001), maximal ferritin level (743.35 (330.7–1600.5) vs. 190.20 (61.2–351.4), p < 0.001) and minimal albumin levels (3.00 (2.5- 3.5) vs. 3.5 (3.10- 4.00) p < 0.001). In accordance with this, hospitalization length (in days) was longer among COVID-19 compared to influenza patients (7.00 (3.00–14.75) vs. 4.00 (2.00–7.00), p < 0.001). Furthermore, COVID-19 patients had profoundly higher intubation (19.2% vs. 4.4%, p < 0.001) and death rates (26.3% vs. 7.0%, p < 0.001).

**Table 1:** Patient’s demographic and clinical characteristics.

	Influenza (N = 724)	SARS-CoV-2 (N = 642)	p-value
Gender—male (%)	340 (47)	380 (59.2)	0.15
Age (median, IQR)	67.00 (43–78)	68.00 (53–78)	0.023
Death (%)	51 (7.0)	169 (26.3)	< 0.001
Intubated (%)	32 (4.4)	123 (19.2)	< 0.001
IORD (%)	71 (9.8)	215 (33.5)	< 0.001
Body mass index (median, IQR)	26.3 (22.7- 30)	27.34 (24–30.9)	< 0.001
Systolic blood pressure_min_ (median, IQR)	110 (97–127)	105 (95–120)	< 0.001
Saturation_min_ (median, IQR)	94 (90–97)	90 (84–94)	< 0.001
Albumin_min_ (median, IQR)	3.5 (3.1–4)	3 (2.5–3.5)	< 0.001
Lymphocytes_min_ (median, IQR)	0.60 (0.4–0.9)	0.60 (0.4–0.9)	0.191
International normalized ratio (INR)_max_ (median, IQR)	1.11 (0.98–1.29)	1.20 (1.10–1.39)	< 0.001
C-reactive protein (CRP)_max_ (median, IQR)	7.05 (3.3–15.4)	13.41 (6.22–24.94)	< 0.001
White blood cells_max_ (median, IQR)	9.14 (6.6–12.4)	10.32 (6.75–15.94)	< 0.001
Ferritin_max_ (median, IQR)	190.2 (61.2–351.4)	743.35 (330.7–1600.5)	< 0.001
Lactate dehydrogenase_max_ (median, IQR)	537.5 (438- 684.7)	737 (545–1093)	< 0.001
**Number of coinfections (%)**			0.006
0	661 (91.3)	561 (87.4)	
1	51 (7)	52 (8.1)	
More than > 1	12 (1.7)	29 (4.5)	
Hospitalization length (median, IQR)	4 (2- 7)	7 (3–14.75)	< 0.001

Data are shown as median (interquartile range (IQR)) or n (%) as indicated.

min, minimal levels; max, maximal levels, IORD, intubation or death.

### Characterization of the bacterial pathogens among influenza and COVID-19 patients

COVID-19 patients had higher rates of secondary bacterial infections than influenza patients (12.6% vs. 8.7%, p = 0.006). To test whether there was also a difference in the nature of the infecting pathogens between the two groups, we further compared the different types of bacteria in each group (Table 2). For this purpose, we defined infections occurring < 48 h after admission as early infections, while infections occurring between 2–14 days post-admission were defined as late infections. Infections occurring after 14 days were disregarded. Interestingly, the types of isolated bacteria were generally similar between COVID-19 and influenza patients. While infections with gram-negative bacteria represented most infections (75%) in both groups, there were no significant differences between the groups neither in the early nor late infections. In contrast, late infections with gram-positive bacteria were more common in COVID-19 than influenza patients (28% vs. 9.5%, p = 0.01).

**Table 2:** Main documented bacterial coinfections according to the type of viral disease.

Variable	Influenza (N = 63)	SARS-CoV-2 (N = 81)	p-value
Gram positive-early	15 (24%)	11 (14%)	0.2
Gram positive-late	6 (9.5%)	23 (28%)	0.010
Gram positive-total	21 (33%)	34 (42%)	0.4
Gram negative-early	26 (41%)	20 (25%)	0.053
Gram negative-late	21 (33%)	41 (51%)	0.056
Gram negative-total	47 (75%)	61 (75%)	> 0.9
*Pseud. aeruginosa*-early	11 (17%)	9 (11%)	0.4
*Pseud. aeruginosa-*late	9 (14%)	11 (14%)	> 0.9
*Pseud. aeruginosa-*total	20 (32%)	20 (25%)	0.5
*E. coli-*early	6 (9.5%)	7 (8.6%)	> 0.9
*E. coli-*late	4 (6.3%)	7 (8.6%)	0.8
*E. coli-*total	10 (16%)	14 (17%)	> 0.9
*Enterobacter-*early	1 (1.6%)	0 (0%)	0.4
*Enterobacter-*late	0 (0%)	5 (6.2%)	0.068
*Enterobacter-*total	1 (1.6%)	5 (6.2%)	0.2
*Acinetobacter-*early	2 (3.2%)	1 (1.2%)	0.6
*Acinetobacter-*late	6 (9.5%)	11 (14%)	0.6
*Acinetobacter-total*	8 (13%)	12 (15%)	> 0.9
*Staph. aureus-*early	7 (11%)	6 (7.4%)	0.6
*Staph. aureus-*late	4 (6.3%)	12 (15%)	0.2
*Staph. aureus-*total	11 (17%)	18 (22%)	0.6
*Proteus mirabilis-*early	0 (0%)	2 (2.5%)	0.5
*Proteus mirabilis-*late	0 (0%)	2 (2.5%)	0.5
*Proteus mirabilis-*total	0 (0%)	4 (4.9%)	0.13
*Enterococcus-*early	0 (0%)	2 (2.5%)	0.5
*Enterococcus-*late	0 (0%)	5 (6.2%)	0.068
*Enterococcus-*total	0 (0%)	7 (8.6%)	0.018
*Coryneb. striatum-*early	4 (6.3%)	1 (1.2%)	0.2
*Coryneb. striatum-*late	0 (0%)	6 (7.4%)	0.035
*Coryneb. striatum-*total	4 (6.3%)	7 (8.6%)	0.8
*K. Pneumoniae-*early	2 (3.2%)	3 (3.7%)	> 0.9
*K. Pneumoniae-*late	0 (0%)	7 (8.6%)	0.018
*K. Pneumoniae-*total	2 (3.2%)	10 (12%)	0.095
*S. marcescens-*early	1 (1.6%)	0 (0%)	0.4
*S. marcescens-*late	0 (0%)	4 (4.9%)	0.13
*S. marcescens-*total	1 (1.6%)	4 (4.9%)	0.4
*Strep. Pneumonia-*early	5 (7.9%)	2 (2.5%)	0.2
*Strep. Pneumonia-*late	2 (3.2%)	1 (1.2%)	0.6
*Strep. Pneumonia-*total	7 (11%)	3 (3.7%)	0.10
*H. influenza-*early	5 (7.9%)	2 (2.5%)	0.2
*H. influenza-*late	2 (3.2%)	5 (6.2%)	0.5
*H. influenza-*total	7 (11%)	7 (8.6%)	0.8
*Stenotr. Maltophilia-*early	1 (1.6%)	0 (0%)	0.4
*Stenotr. Maltophilia-*late	3 (4.8%)	6 (7.4%)	0.7
*Stenotr. Maltophilia-*total	4 (6.3%)	6 (7.4%)	> 0.9
*Actinomyces-*early	1 (1.6%)	1 (1.2%)	> 0.9
*Actinomyces-*late	1 (1.6%)	2 (2.5%)	> 0.9
*Actinomyces-*total	2 (3.2%)	3 (3.7%)	> 0.9
Other-early	1 (1.6%)	1 (1.2%)	> 0.9
Other-late	1 (1.6%)	2 (2.5%)	> 0.9
Other-total	2 (3.2%)	3 (3.7%)	> 0.9
Hospitalization length	8 (4, 21)	12 (6, 23)	0.10
Days to first bacteria	1.0 (1.0, 3.0)	4.0 (1.0, 8.0)	< 0.001

Most secondary bacterial infections in both groups were derived from blood cultures (supplementary table 1).

A notable difference was observed in the late gram-positive infections. In COVID-19 patients, 85% of these infections were isolated from blood cultures, while only 14.2% were isolated from respiratory samples. In contrast, among influenza patients, the isolated bacteria divided equally between blood and respiratory-derived cultures.

Notably, the overall time from admission to bacterial growth was longer in COVID-19 compared to influenza patients (4 (1–8) vs. 1 (1–3) days, p < 0.001).

The most common bacterial coinfection in both groups was *Pseudomonas aeruginosa* (32% vs. 25% of influenza and COVID-19, respectively, p = 0.5) and *Staphylococcus aureus* (17% vs. 22%, respectively, p = 0.6). Notably, a significant difference was observed in the percentage of *Enterococcus* infections between influenza and COVID-19 (0% vs. 8.6%, p = 0.018). Significant differences were observed in late *Corynebacterium striatum* and *Klebsiella pneumoniae* infections, as well.

### Parameters associated with death among influenza and COVID-19 patients with bacterial coinfection

We next tested major clinical and epidemiological variables associated with death in the infected population from both groups, using univariate logistic analysis (Table 3). Only age 1.01-fold (1.00–1.02), p = 0.013) and maximal international normalized ratio (INR, a unified clotting measure) 3.83-fold (2.34–6.65), p < 0.001) were associated with death in COVID-19 but not in influenza patients (age odds ratio (OR) 1.00 (0.99–1.01), p = 0.6, OR 1.34 (0.94–1.85), p = 0.071, for age and INR, respectively).

**Table 3:** Univariate analysis of variables associated with death.

	Influenza (n = 724)			COVID-19 (n = 642)		
	OR (95% CI)		p-value	OR (95% CI)		p-value
**Bacteria growth (time)**						
Early < 48 h	3.09 (1.11–7.38)		0.018	2.70 (1.22–5.83)		0.012
Late > 48 h	4.78 (1.67–12)		0.002	7.00 (3.88–13.0)		< 0.001
Coinfection count	2.08 (1.3–3.21)		0.001	2.52 (1.86–3.54)		< 0.001
**Number of coinfections**						
1	3.42 (1.47–7.26)		0.002	3.33 (1.86–5.96)		< 0.001
> 1	5.32 (1.15–18.7)		0.015	11.3 (4.95–29.2)		< 0.001
Hospitalization length_days_	1.04 (1.02–1.06)		< 0.001	1.02 (1.01–1.04)		0.003
Gender (male)	1.29 (0.73–2.30)		0.4	1.31 (0.91–1.89)		0.15
Age	1.00 (0.99–1.01)		0.6	1.01 (1.00–1.02)		0.013
Body mass index (BMI)	1.00 (1.00–1.00)		> 0.9	1.00 (1.00–1.00)		0.7
Systolic blood pressure_min_	0.93 (0.92–0.95)		< 0.001	0.95 (0.94–0.96)		< 0.001
Blood oxygen saturation_min_	0.88 (0.84–0.91)		< 0.001	0.87 (0.85–0.90)		< 0.001
Fever_max_	1.74 (1.26–2.44)		< 0.001	1.48 (1.22–1.82)		< 0.001
Albumin_min_	0.13 (0.08–0.21)		< 0.001	0.12 (0.08–0.17)		< 0.001
Lymphocytes_min_	0.15 (0.05–0.37)		< 0.001	0.15 (0.09–0.26)		< 0.001
International normalized ratio_max_	1.34 (0.94–1.85)		0.071	3.83 (2.34–6.65)		< 0.001
C-Reactive protein_max_	1.07 (1.05–1.1)		< 0.001	1.09 (1.07–1.11)		< 0.001
Total Bilirubin_max_	1.58 (1.29–2)		< 0.001	1.61 (1.37–1.93)		< 0.001
White blood cells_max_	1.05 (1.02–1.09)		0.010	1.12 (1.09–1.15)		< 0.001
Neutrophils_max_	1.12 (1.07–1.17)		< 0.001	1.15 (1.12–1.18)		< 0.001
Ferritin_max_	1.00 (1.00–1.00)		0.026	1.00 (1.00–1.00)		< 0.001
Lactate dehydrogenase_max_	1.00 (1.00–1.00)		< 0.001	1.00 (1.00–1.00)		< 0.001
Number of coinfections (first 48 h)	1.92 (0.96–3.44)		0.037	1.84 (1.11–3.22)		0.022
Number of coinfections (2–14 days)	3.20 (1.45–6.59)		0.002	2.91(2.00- 4.46)		< 0.001
Days to First bacteria growth	1.00 (0.90–1.10)		> 0.9	0.97 (0.93–1.02)		0.3

OR, odds ratio; CI, confidence interval; min, minimal levels; max, maximal levels.

Notably, almost all bacterial growth parameters tested were associated with an increased risk of death in both groups. Both early infection 3.09-fold (1.11- 7.38) p = 0.018 for influenza and 2.70-fold (1.22- 5.83) p = 0.012 for COVID-19), and late infection (4.78-fold (1.67- 12.0) p = 0.002 for influenza, and 7.00-fold (3.88–13.0) p < 0.001 for COVID-19) were associated with an increased risk of death, with late infections being associated with a higher risk. The total number of infections was also related to an increased risk of death in both COVID-19 (3.33-fold (1.86–5.96), p < 0.001 for one infection and 11.3-fold (4.95–29.2), p < 0.001 for more than one infection) and influenza (3.42-fold (1.47–7.26), p = 0.002 for one infection and 5.32-fold (1.15–18.7), p = 0.015 for more than one infection). The number of early infections (COVID-19, 1.84-fold (1.11–3.22), p = 0.022); influenza 1.92-fold (0.96–3.44), p = 0.037) and late infections (COVID-19, 2.91-fold (2.00- 4.46), p < 0.001); influenza 3.20-fold (1.45–6.59), p = 0.002) were also associated with an increased risk of death, with the later infections showing higher risk levels.

**Figure 1:**
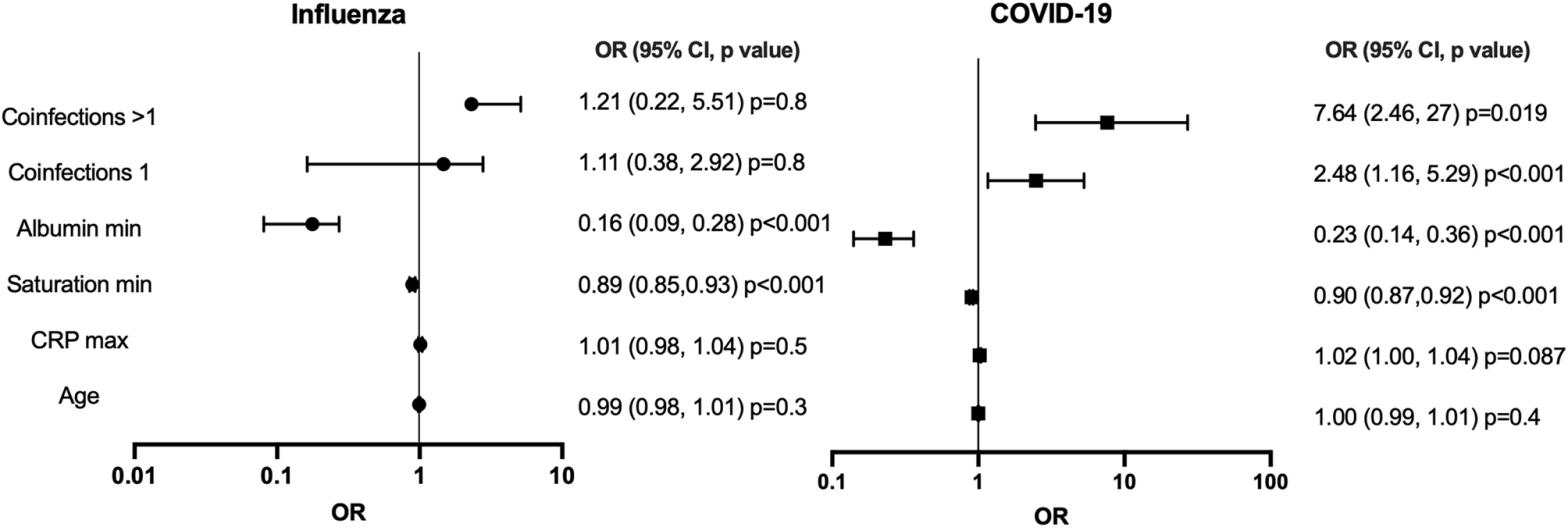
Multivariate analysis of various parameters related to disease severity and their association with death. For each viral infection, the odds ratios (OR) for each parameter and 95% confidence intervals (CI) are plotted.

The association of the number of secondary bacterial infections with death remained significant upon adjustment to age, minimal blood oxygen saturation, maximal CRP and minimal albumin levels, all variables of disease severity, in COVID-19 (one infection 2.48-fold (1.16–5.29), p = 0.019; more than one infection 7.64-fold (2.46–27), P < 0.001)**,** but not in the infected influenza patients (one infection 1.11-fold (0.38–2.92), p = 0.8; more than one infection 1.21-fold (0.22–5.51), P = 0.8) (Fig. 1).

**Figure 2:**
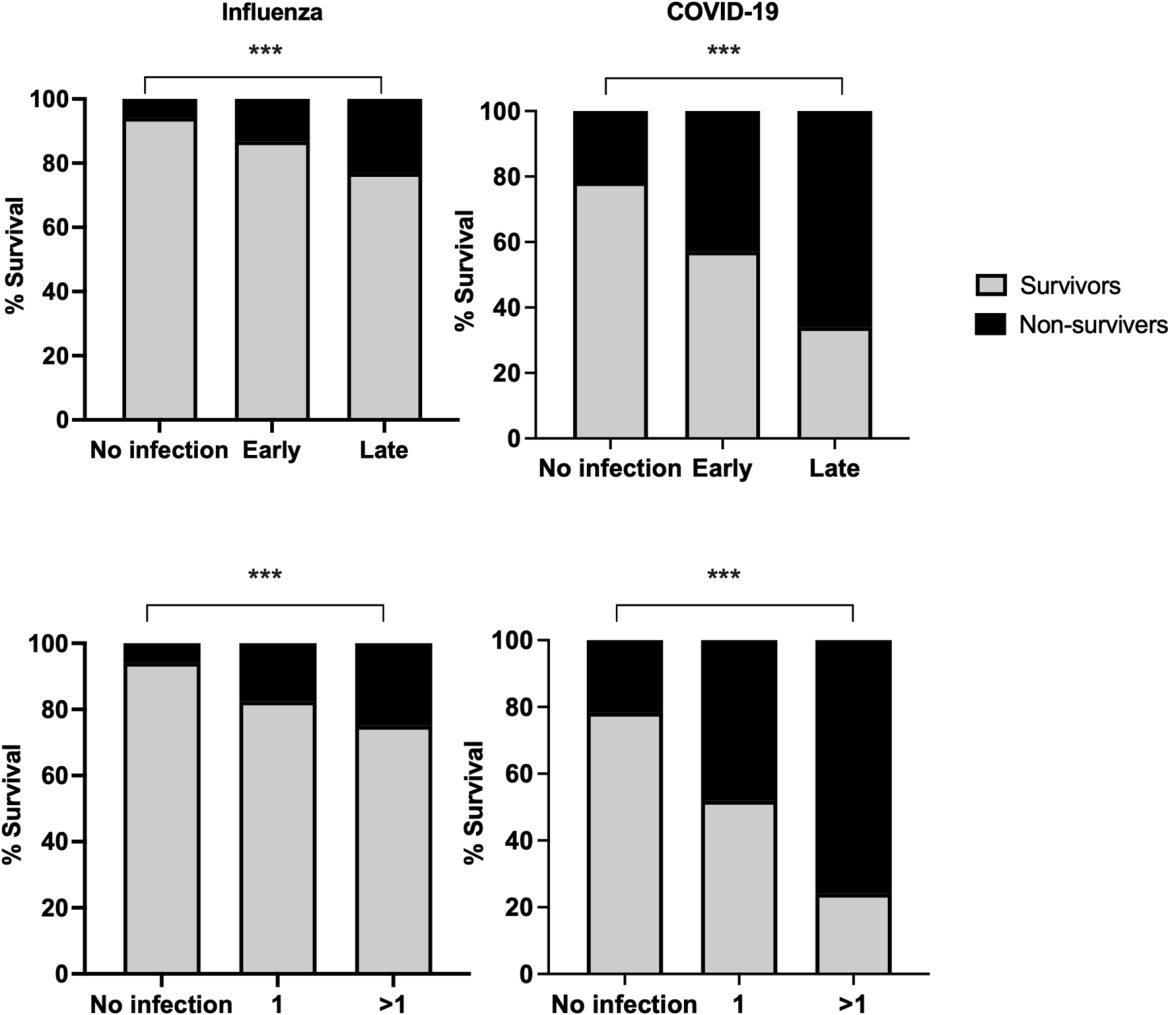
Association of coinfection variables with disease outcome. Stacked graphs are presenting the percentage of surviving or non-surviving coinfected patients in both groups. The top graphs compare survival rates in each group according to the time of infection, while the lower graphs compare the survival rates according to the number of infections. Chi-Square Test was used to compare the categorical variables, ***, p < 0.001.

Last, we calculated the percentage of deaths with respect to the presence or absence of secondary bacterial infections in the COVID-19 and Influenza groups (Fig. 2). Bacterial infections resulted in significantly decreased survival rates in both groups. While 13.2% of patients without infections died, 33% of patients with one infection and 61% of patients with two or more infections died (Chi-Square Test, no infections p < 0.001, one infection p = 0.001, more than one p = 0.002).

Comparison between the influenza and COVID-19 groups showed that patients infected with COVID-19 have a higher risk of death. In the influenza group 17.6% of patients with one infection, and 25% of the patients with more than one infection died. While in the COVID-19 group, 48.1% of patients in the group with one infection, and 75.9% of patients with more than one infection died (Chi-Square Test, influenza p < 0.001, COVID-19 p < 0.001). A similar analysis was performed regarding the time of the infection in both COVID-19 and influenza groups. In the group of patients without bacterial infection 13.2% of the patients died while 27.7% of patients with early infection and 51.9% of patients with late infections died (Chi-Square Test, no infection p < 0.001, early p < 0.001, late p < 0.001). Comparison between the influenza and COVID-19 groups indicated that late infections in COVID-19 patients resulted in higher death rates. In the influenza group, 5.9% of patients with no infection died. The death rates were 16.2% and 23.1% among patients with early or late infections, respectively. Among COVID-19 patients, 21.7% of patients died in the group with no bacterial infection. The death rates were 42.9% and 66% among patients with early or late infections (Chi-Square Test, influenza p < 0.001, COVID-19 p < 0.001).

## Discussion

In this study, we compared two large cohorts of influenza, and COVID-19 patients admitted to a large tertiary center and from which bacterial cultures from blood, sputum, and/or BAL were obtained. Most secondary bacterial infections in both groups were derived from blood cultures. Patients infected with these viruses suffer from acute respiratory distress; therefore, clinicians mostly avoid taking sputum and BAL tests. This might affect the number of infections identified in blood cultures vs. cultures from respiratory origins. However, it should be also taken into account that respiratory infections might become systemic with time as well. In general, COVID-19 patients were more severely ill, as reflected by various disease severity markers, and had worse outcomes, reflected by a higher percentage of intubation or death than influenza patients. Importantly, COVID-19 patients had more documented secondary bacterial infections than influenza patients, and these infections were independently associated with death in COVID-19 but not in influenza patients.

These findings suggest that secondary bacterial infections might be a significant, potentially treatable, contributing factor for disease severity among COVID-19 patients.

Our results are in line with previous studies reporting that COVID-19 patients suffer from a more severe disease and ~ 3 times higher death rates) than influenza patients. In addition, in-hospital death of patients with pulmonary secondary bacterial infection was two-times higher in COVID-19 patients^8^.

In this study, the most common bacterial infections in patients with either influenza or COVID-19 were *Pseudomonas aeruginosa* and *Staphylococcus aureus*, generally in agreement with previous studies^8^. *Staphylococcus aureus* is a known pathogen associated with secondary pneumonia during influenza infection^9^. Its dissemination to the lungs is attributed to a combination of environmental changes and immune responses that create suitable conditions for *Staphylococcus aureus* infection^9^. *Pseudomonas aeruginosa* is also associated with chronic predisposing respiratory conditions, including upper respiratory tract infections such as influenza^10,11^. While *Pseudomonas aeruginosa* is a common respiratory opportunistic pathogen, it is also known as the most common gram-negative bacterial species associated with severe hospital-acquired infections in some hospitals^12^.

Interestingly, a significant difference was observed in the percentage of *Enterococcus* infections between influenza and COVID-19 (0% vs. 8.6%, p = 0.018). Furthermore, late infections with gram-positive bacteria were more common in COVID-19 compared to influenza patients. Of note, a large multi-center study from Sweden comparing bacterial growth in 15,103 blood cultures from COVID-19 patients with non-infected controls also found that the rate of infections with gram-positive bacteria was significantly higher in patients with COVID-19 (66% vs. 50%, p < 0.0001)^13^.

Generally, the overall time from admission to bacterial growth was longer for COVID-19 compared to influenza patients. This possibly reflects the disease's natural history, characterized by a late deterioration, typically 7–10 days after symptoms onset, that might be accompanied by a secondary bacterial infection. Alternatively, COVID-19 patients are admitted in isolated dedicated wards under strict isolation protocol, limiting free medical and nursing personnel access. This might affect the quality of medical treatment and increase the likelihood of complications such as nosocomial infections, including line, device-related, skin, and soft tissue infections associated with gram-positive bacteria^14^. This hypothesis is in line with the increased hospitalization length, the longer overall time from admission to bacterial growth, and the higher rates of late gram-positive bacterial infections observed in COVID-19 than influenza patients. In addition, the higher fraction of late gram-positive infections isolated from blood cultures in COVID-19 compared to influenza, further supports this notion.

Although several studies characterized the secondary bacterial infections in COVID-19 patients, our study is the first to compare the infecting bacterial pathogens with those observed in influenza patients from the same center, and to correlate secondary bacterial infections in both groups with disease severity and outcome.

Our study, however, has several limitations. First, it is retrospective in nature and relies on proper documentation of cultures and clinical parameters in the medical records. The study might also suffer from selection bias since the decision to obtain bacterial cultures was done by the treating clinician and most probably was affected by the severity of the disease. Second, given the local screening policy of every admitted patient for COVID-19 during the pandemic, we also cannot rule out that a fraction of patients (especially among the COVID-19 group) was hospitalized due to other medical conditions implicated in bacteremia that were completely unrelated to their viral infection. Furthermore, since there were no influenza cases in Israel during the COVID-19 pandemic, the two groups of patients might reflect different time periods. Last, the study's data was from a single center, and thus the infecting bacteria might reflect a site-specific microbiological profile.Taken together, our results show that secondary bacterial infections, in particular late gram-positive infections, are a clinically important complication with a significant correlation to poor outcomes in hospitalized COVID-19 patients. This calls for increased awareness of the treating physicians to the possibility of secondary bacterial infection as an etiology to late deterioration, suggesting that antibiotic treatment may be an essential component of the therapeutic armamentarium in selected patients with severe COVID-19. As gram-positive bacteria are increasingly becoming resistant to antibiotics^15^, our results highlight the importance of implementing infection control measures specific for COVID-19 hospitalized patients in addition to modification of antibiotic treatment protocols.

## Methods

### Study design, data collection, and outcomes

Influenza and COVID-19 patients admitted to Rabin Medical Center, a large tertiary center in Israel, between October 2018 to February 2021 were selected for the study. Viral infection was confirmed using a specific RT-PCR assay performed on nasopharyngeal and throat swabs (COPAN Diagnostics Inc.). SARS-CoV RT-PCR was performed using the Allplex™ 2019-nCoV Assay (Seegene). Influenza detection was performed using Xpert®Xpress Flu/RSV (Cepheid) or the Allplex™ Respiratory Panel 1 (Seegene). A total of 2146 admitted patients diagnosed with Influenza (n = 923) or COVID-19 (n = 1223) were identified according to their medical files. The need for informed consent was waived due to the retrospective nature of the study. Only patients for whom blood and/or sputum/bronchoalveolar lavage (BAL) cultures were taken upon admission or during hospitalization were included in the study. After excluding the patients for which no culture was taken, 742 influenza and 642 COVID-19 patients were selected for further analysis.

Blood or sputum cultures were taken routinely at admission or according to the discretion of the treating clinician. The samples were transferred to the microbiology laboratory and processed according to the current standard practice procedures^16^. Sputum or BAL specimens were cultured, some of the samples were also tested using BIOFIRE® FILMARRAY® Pneumonia plus Panel according to the discretion of the treating clinician. Blood cultures were analyzed using the BACTEC™ blood culture system /BACTEC™ FX system (Becton Dickinson, Inc). Positive cultures were further analyzed according to the current standard practice procedures^16^. Identification of isolates was performed using matrix-assisted laser desorption ionization–time-of-flight mass spectrometry. GeneXpert assay was performed directly on positive blood cultures suspected of containing staphylococci after the gram staining.

Bacterial growth during the first 48 h from admission was regarded as early or coinfection, and bacterial growth up to 2 weeks from admission was defined as a late infection. The study was approved by Rabin Medical Center Review Board (#RMC-20–0142) and the Tel Aviv University Ethics Committee (0001269–3) and was performed according to the Helsinki declaration.

### Statistical analysis

The patient’s cohort was divided into two groups based on the viral infection diagnosis (influenza and SARS-CoV-2). Normality tests were conducted for all variables. Due to the non-normal distribution of our variables, we used the non-parametric Wilcoxon and Kruskal Wallis tests when appropriate, and median and interquartile range (IQR) are presented. Chi-square or Fisher’s exact tests were used to compare categorical variables between study groups accordingly, p < 0.05 (two-tailed test) was considered statistically significant. Univariate and multivariate regression models were constructed to estimate predictors for the primary outcome. ORs with 95% confidence intervals (CI) were calculated. Logistic regression was used to calculate ORs. Statistical analysis was performed using SPSS 25, R 4.04, and GraphPad PRISM 8.

## Supplementary Information

Supplementary Information.
