# Supplementary material for: Secondary bacterial infection in COVID-19 patients is a stronger predictor for death compared to influenza patients
Source: Sci Rep. 2021 Jun 16;11:12703. doi: 10.1038/s41598-021-92220-0 (PMC8209102; doi:10.1038/s41598-021-92220-0)
Supplement: Supplementary file 1 — Supplementary Information. [file 41598_2021_92220_MOESM1_ESM.docx]

**Supporting information for the article “Secondary bacterial infection in COVID-19 patients is a stronger predictor for death compared to influenza patients”.**

**Supplementary Table 1: Types of bacterial co-infections and their source of isolation in influenza and COVID-19 patients.**

|  | Influenza | | COVID-19 | | |
| --- | --- | --- | --- | --- | --- |
|  |  | Early | Late | Early | Late |
| Gram-positive | Blood | 82% | 50% | 83.30% | 85.7% |
|  | Sputum | 17.6% | 50% | 16.60% | 14.2% |
|  | BAL | 0 | 0 | 0 | 0 |
| Gram-negative | Blood | 90% | 68.90% | 73% | 85.9% |
|  | Sputum | 10% | 31% | 19.20% | 14% |
|  | BAL | 0% | 0% | 7.69% | 0.0% |
